# Utilization of egg-laying hens (*Gallus Gallus domesticus*) for production of ingredients for human consumption and animal feed

**DOI:** 10.1186/s12896-020-00618-x

**Published:** 2020-05-06

**Authors:** Veronica Hjellnes, Rasa Šližyte, Turid Rustad, Ana Karina Carvajal, Kirsti Greiff

**Affiliations:** 1 Department of Biotechnology and Food Science, Norwegian University of Science and Technology, NTNU, 7491 Trondheim, Norway; 2 SINTEF Sealab, Brattørkaia 17 C, 7010 Trondheim, Norway

**Keywords:** Rest raw material, Thermal treatment, Enzymatic hydrolysis, Silaging, Lipid, Protein, Food and feed ingredients, Sustainable utilization of food resources

## Abstract

**Background:**

In Norway, 3 million discarded egg-laying hens are destructed annually, which equals 1500 tons pure hen meat. Due to the slaughter methods used, this raw material is handled as a high-risk waste, while in reality it constitutes a source of valuable components like proteins and lipids.

**Methods:**

This study assess different processing methods (thermal treatment, enzymatic hydrolysis and silaging) for utilization of discarded egg-laying hens for the production of ingredients for human consumption and animal feed. The processing methods were evaluated on the basis of quantity and quality of the obtained products.

**Results:**

Thermal treatment and enzymatic hydrolysis resulted in extraction of good quality lipids from the raw material. The separated oil (50.1–82.3% of the total lipid content in the raw material) was of high quality based on the content of free fatty acids (≤ 1.0%) and total oxidation value (≤ 3.9). Enzymatic hydrolysis also enabled separation of protein in the form of protein hydrolysate. Addition of Protamex and Papain+Bromelain significantly (*p* ≤ 0.05) increased the protein content (85.1–94.6%) and decreased the lipid content (0.3–1.1%) in the hydrolysate compared to autolysis (protein content: 64.8–72.3%, lipid content: 1.0–2.6%). Silaging increased the protein digestibility (63.2–79.7% compared to 57.3–66.2% for untreated raw material), and thus constitutes a good method for utilizing the protein content of the raw material for animal feed.

**Conclusion:**

The biotechnological processing methods thermal treatment, enzymatic hydrolysis and silaging can be used to increase the utilization of discarded egg-laying hens for production of ingredients for human consumption and animal feed.

## Background

Egg-laying hens play an important role in traditional Norwegian agriculture. A large amount of egg is produced to meet consumer demands, with an estimated consumption of 12.5 kg eggs per person per year [1]. At the age of about 18 months the egg-laying hens no longer produce a sufficient amount of eggs, and are discarded. It is challenging to utilize the hen meat in traditional slaughter lines due to the difference in morphology and chemical composition between egg-laying hens and broiler chickens. Only a small amount of egg-laying hens are currently being used as food, while the rest of this raw material is treated as high-risk waste. Three million discarded egg-laying hens are destructed annually in Norway, which equals the amount of 1500 tons pure hen meat [2]. In addition to the ethical issues associated with such handling of a potential food, the destruction also constitutes an economic burden for the egg farmers.

The global increase in population results in an increased demand for food, in particular foods containing proteins of high nutritional value [3]. Additionally, there is a lack of protein sources for animal feed production in Norway [4]. This requires a better utilization of all available resources. Furthermore a lot of effort has been put into studying the possibility of utilizing by-products from the food industry in Europe [5]. Processed in a suitable manner, by-products can be a source of valuable nutrients to meet the increased demand for food and feed [6]. The same goes for egg-laying hens, which are currently treated as a waste from the egg-production industry in Norway. Despite being handled as a high-risk waste, egg-laying hens represents raw material with a valuable chemical composition. This raw material can be used directly in food, or act as a source of valuable nutritional components like proteins and lipids.

Thermal treatment, enzymatic hydrolysis and silaging are well known and simple technological processing methods for biological raw material. The processing conditions used in this research were originally developed for utilization of marine by-products, and it was of interest to see if they could be applicable for processing of discarded egg-laying hens as well. Thermal treatment (rendering) can be used to separate lipids from the raw material [7]. The temperature and duration of the thermal treatment will influence the amount and quality of the separated oil [8]. Enzymatic hydrolysis is a processing method where the enzymatic activity of endogenous (autolysis), or added commercial proteases (accelerated hydrolysis), is utilized to hydrolyze the proteins in the raw material. The solubilized peptides end up in the main product of the enzymatic hydrolysis: the protein hydrolysate [9]. Beside solubilizing, hydrolysis might also improve the nutritional and functional characteristics of the proteins [10]. The hydrolysate can be used as a functional ingredient in food, as a water-binder, flavor-binder or simply to increase the protein content of the food in question [11]. Silaging, like thermal treatment, constitutes a traditional, low cost and simple processing method [12]. In silaging, the pH of the raw material is lowered to stabilize the raw material and facilitate the activity of endogenous proteases, mainly pepsin. The main product of silaging, the silage, is a liquid mass of hydrolyzed proteins and lipids which can serve as a protein source in animal feed [9, 13]. The purpose of this study was to assess different methods for processing of discarded egg-laying hens for the production of ingredients for human consumption and animal feed. The main goal was to obtain proteins and lipids by using the mentioned technologies, and evaluate how the different processing parameters influence yield and quality of the obtained fractions.

## Methods

### Raw material

Discarded egg-laying hens (*Gallus Gallus domesticus*) of the breed *Leghorn* were provided by the slaughterhouse of Ytterøykylling AS (Ytterøy, Norway). The hens were slaughter at approved facilities following the Norwegian Food Production and Food Safety Act [14] and Animal Welfare Act [15] prior to transport to lab facilities for experimental analyses. Two different groups of hen raw material were used for this work. One group consisted of hens with viscera, head and feathers (**H**en **w**ith, Hw), while another consisted of hens without viscera, head and feathers (**H**en **w**ith**o**ut, Hw/o).

Fresh raw material was minced (HOBART model AE 200) using hole size 10 mm, followed by 5 mm, to a homogenous mass. The minced raw material was packed in fractions of about 2 kg, frozen and stored at − 80 °C. Before use, the raw material was thawed overnight at 4 °C.

### Enzymes and chemicals

Protamex, manufactured by Novozymes A/S (Bagsvaerd, Denmark), Corolase PP, manufactured by AB Enzymes (Darmstadt, Germany), Papain and Bromelain, manufactured by Enzybel International (Waterloo, Belgium), acetic acid, formic acid, sodium bisulfite, methanol, chloroform, hexane, formaldehyde, iso-octane and pepsin (Merck, Darmstad, Germany) were used for the experiments and chemical analysis. Boron Trifluoride-Methanol solution (BF3-methanol, Sigma, St. Louis, MO, USA) was used in pre-analytic preparation of fatty acid methyl esters.

### Processing methods

**Fig. 1:**
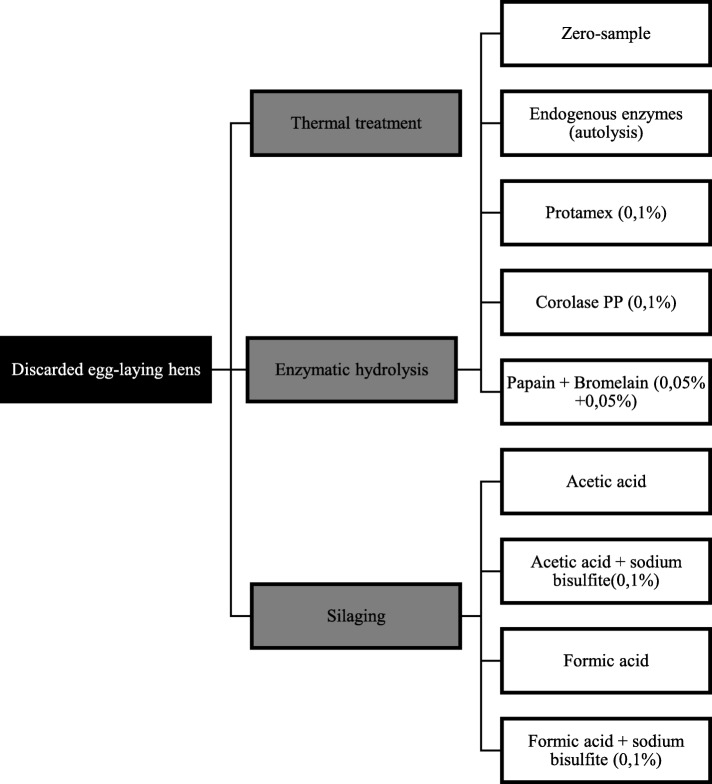
Screening experiment. Schematic overview of the screening experimental setup for processing of discarded egg-laying hens by thermal treatment, enzymatic hydrolysis and silaging

Three different processing methods were used to obtain lipids and proteins from the raw material: thermal treatment, enzymatic hydrolysis and silaging. An overview of the screening experiment is presented in Fig. 1. All processing were conducted in 50 mL centrifuge tubes.

#### Thermal treatment

Raw material was transferred to centrifuge tubes, 40 g in each. The samples were heated in microwave (750–800 W) to a temperature of 70–80 °C, before being placed in a water bath (100 °C) for 15 min of thermal treatment (T, natural pH). After processing, all tubes were centrifuged for 10 min (3075×g, 40 °C), then frozen in an upright position and stored at − 80 °C.

The experiment was performed in 10 parallels for both raw materials, hereafter referred to as HwT and Hw/oT respectively.

#### Enzymatic hydrolysis

Raw material was mixed (1:1) with warm water (50 °C). The mixture was heated in microwave (750–800 W) to a temperature of 50 °C before addition of commercial enzymes. The mixture was transferred to centrifuge tubes, 40 g in each. The centrifuge tubes were attached to a rotator (Rotator SB3, Stuart Equipment), and placed in an incubator (50 °C) for the hydrolysis. Enzymatic activity was terminated by heat inactivation, which was carried out by heating the samples in microwave (750–800 W) to a temperature of 90 °C, and kept for 10 min. After processing, all tubes were centrifuged for 10 min (3075×g, 40 °C), frozen in an upright position and stored at − 80 °C.

Enzymatic hydrolysis (H) was conducted on minced raw material at 50 °C for 60 min (natural pH). The experiment was performed in 10 parallels per treatment for both raw materials, hereafter referred to as HwH and Hw/oH respectively. Five different treatments were performed in the enzymatic hydrolysis (Fig. 1): without hydrolysis (zero-sample, 0), hydrolysis with endogenous enzymes (autolysis, A), addition of 0,1% Protamex (Pr), addition of 0,1% Corolase PP (PP) and addition 0,1% of a 1:1 mixture of Papain + Bromelain (P + B).

#### Silaging

The pH of the raw material was reduced to pH = 3 by dropwise addition of acid. Sodium bisulfite (0,1%) was then added to part of the mixtures. The raw material was transferred to centrifuge tubes, 40 g in each, sealed with parafilm and placed in an incubator (40 °C) for 24 h. Enzymatic activity was terminated by heat inactivation, which was carried out by heating the samples in microwave (750–800 W) to a temperature of 90 °C, and kept for 10 min. After processing, all tubes were centrifuged for 10 min (3075×g, 40 °C), frozen in an upright position and stored at − 80 °C.

Silaging (S) was conducted on minced raw material at 40 °C for 24 h (pH = 3). The experiment was performed in 5 parallels per treatment for both raw materials: Hw and Hw/o, hereafter referred to as HwS and Hw/oS respectively. Two different acids were used to reduce the pH: acetic acid (A) and formic acid (F). Sodium bisulfite (Su) was added to assess its antioxidative ability. This gave four different treatments, illustrated in Fig. 1.

#### Fractionation of the samples

The different fractions (oil, soluble protein and sediment) were separated by cutting the material while frozen. All fractions were weighed, and individually sampled for dry matter and ash analysis. Parallels of the same fractions were merged, frozen and stored at − 80 °C. Protein hydrolysates were freeze dried before analysis.

### Analysis of the raw material and processing products

Dry matter and ash content were analyzed by gravimetric analysis. Ash content was estimated according to AOAC [16]. The samples were analyzed in triplicates to quintuplicates.

Total nitrogen was determined by Carlo-Erba NA-1500 CN-analyzer (Carlo Erba Instruments S.pA., Milan, Italy). Crude protein was estimated by multiplying total N with a factor of 6.25. The samples were analyzed in triplicates to quadruplicates.

Total lipid content was estimated by lipid extraction according to the method of Bligh & Dyer [17]. The samples were analyzed in duplicates to triplicates.

Pepsin digestibility was determined as described in AOAC 971.09 [16] using defatted samples. The fat was extracted based on the principle of Bligh & Dyer [17]. A mixture of chloroform and methanol (1:1) was added to wet sample, mixed and centrifuged for 5 min (2000 x g, 10 °C). After removal of solvent, the samples were dried overnight in fume hood. The samples were analyzed in duplicates.

### Analysis of the oil fraction

The amount of free fatty acids (FFA) was determined as % oleic acid, as described by Bernardez et al. [18] with modifications. Iso-octane was used as solvent instead of cyclo-hexane. The samples were analyzed in quadruplicates to octuplicates.

The peroxide value was determined, as described in ISO 3960, based on the AOCS Official Method Cd 8b-90 [19]. The analysis was conducted using half the amount of solvent, and automatic iodometric titration with potentiometric determination of the equivalence point. The samples were analyzed in quadruplicates to octuplicates.

The anisidin value (AV) was determined according to the AOCS Official Method Cd 18–90 [19]. The samples were analyzed in quadruplicates.

TOTOX value was calculated as (2 × *PV*) + *AV*.

The fatty acid composition was determined, using gas chromatography (Agilent 7890 GC System) connected to a Flame ionizing detector (GC-FID), as described by Daukšas et al. [20]. The procedure included pre-analytic methylation of fatty acid. An Agilent CP7713 column (25 m × 250 μm × 0.2 μm), a fatty acid standard (68D, 79, 411, NuChecPrep) and an internal standard (IS, C20:0) was used for the analysis. The samples were analyzed in duplicates.

### Analysis of the protein hydrolysate

The degree of hydrolysis in freeze dried hydrolysate was determined using Formol titration, as described by Taylor [21]. The samples were analyzed in duplicates and triplicates.

The molecular weight distribution of peptides in freeze dried hydrolysate was analyzed using High Performance Liquid Chromatography (HPLC), as described by Hjellnes [22]. The samples were analyzed in duplicates.

### Statistical analysis of data

Microsoft Excel (Microsoft Office 2016) was used for data processing and statistical analyses. Results are reported as average values $\left(\overline{\mathrm{x}}\right)$ of [n] number of parallel ± standard deviation (SD). The T-test was used for comparison of means. Significance level was set to *p* ≤ 0.05.

## Results

### Yield of the fractions after different treatments

The chemical composition of the raw material was analyzed to evaluate the yield of different fractions after processing. The two different raw materials (Hw and Hw/o) varied in chemical composition (Table 1). Raw material Hw had a significantly (Two-sample T(4) = 8.2, p<0.05) higher lipid content (26.9 ± 1.6 g/ 100 g raw material) compared to raw material Hw/o (18.7 ± 1.2 g/ 100 g raw material). This indicates that the viscera of egg-laying hens contain considerable amounts of lipid, an assumption that was confirmed by unpublished data from SINTEF Ocean AS reporting a lipid content of 30% in viscera from egg-laying hens. The higher lipid content of raw material Hw is reflected in the significantly (Two-sample T(4) = 2.9, p<0.05) higher dry matter content (44.0 ± 3.1 g/ 100 g raw material) compared to raw material Hw/o (39.8 ± 1.8 g/ 100 g raw material). The ash content was higher in raw material Hw/o (5.4 ± 1.9 g/ 100 g raw material) compared to raw material Hw (4.9 ± 1.9 g/ 100 g raw material), most likely a result of higher percentage of bones in raw material Hw/o.

**Table 1:** Chemical composition of the raw material

Raw material	Dry matter	Lipid	Protein	Ash
Hw	44.0 ± 3.1	26.9 ± 1.6	18.6 ± 4.5	4.9 ± 1.9
Hw/o	39.8 ± 1.8	18.7 ± 1.2	18.5 ± 4.1	5.4 ± 1.9

Chemical composition of the two raw materials: egg-laying hens with viscera, head and feathers (Hw) and egg-laying hens without viscera, head and feathers (Hw/o). Dry matter, lipid, protein and ash content is given as g/ 100 g raw material ($\overline{\mathrm{x}}$ ± SD, n = 3)

Processing by thermal treatment, enzymatic hydrolysis and silaging fractionated the valuable components (lipid and protein) of the raw material. Thermal treatment gave an oil fraction in addition to a compact sludge, enzymatic hydrolysis gave an oil fraction, a water-soluble protein fraction (hydrolysate) and a compact sludge, while silaging gave an oil fraction and a viscous sludge (silage). The dry matter yield of the different fractions obtained from the processing is presented in Table 2. In addition to the fractions mentioned in Table 2, very low amounts of dry matter from thermal treatment and silaging ended up in the water-soluble fraction.

**Table 2:** Distribution of dry matter after processing

Sample	Oil	Hydrolysate	Sludge
Thermal treatment			
HwT	15.7 ± 0.6		28.3 ± 3.0
Hw/oT	9.4 ± 0.5		25.6 ± 2.5
Enzymatic hydrolysis			
HwH0	18.3 ± 8.3	1.3 ± 0.3	26.5 ± 6.8
HwHA	18.4 ± 10.1	1.5 ± 0.2	23.3 ± 5,4
HwHPr	19.4 ± 5.1	4.9 ± 0.4	18.9 ± 2.3
HwHPP	16.0 ± 5.5	2.6 ± 0.6	21.7 ± 3.8
HwHP+B	22.1 ± 5.9	5.2 ± 0.5	15.8 ± 2.8
Hw/oH0	10.2 ± 2.4	1.6 ± 0.1	26.1 ± 3.3
Hw/oHA	10.1 ± 2.5	1.5 ± 0.2	27.2 ± 4.4
Hw/oHPr	11.2 ± 1.0	6.2 ± 0.6	17.7 ± 1.1
Hw/oHPP	9.6 ± 1.3	4.5 ± 1.2	21.8 ± 4.6
Hw/oHP + B	13.0 ± 2.0	5.5 ± 0.4	19.2 ± 4.2
Silaging			
HwSA	14.3 ± 1.3		24.1 ± 5.1
HwSA.Su	15.6 ± 0.1		20.1 ± 2.9
HwSF	17.7 ± 0.7		19.2 ± 0.0
HwSF.Su	17.4 ± 0.6		17.6 ± 0.0
Hw/oSA	8.0 ± 2.0		25.0 ± 3.4
Hw/oSA.Su	7.4 ± 3.7		25.6 ± 0.0
Hw/oSF	7.0 ± 1.4		30.6 ± 0.0
Hw/oSF.Su	2.6 ± 1.7		28.7 ± 0.0

Dry matter yield in fractions obtained from processing of egg-laying hens with viscera, head and feathers (Hw), and egg-laying hens without viscera, head and feathers (Hw/o), by thermal treatment (T), enzymatic hydrolysis (H) and silaging (S). Abbreviations are given for the individual treatments: without hydrolysis (0), autolysis (A), 0,1% Protamex (Pr), 0,1% Corolase PP (PP), 0,1% Papain + Bromelain (P + B), acetic acid (A), acetic acid + 0,1% sulfite (A.Su), formic acid (F) and formic acid + 0,1% sulfite (F.Su). Dry matter yield is given as dry matter (g)/ 100 g raw material (wet weight) ($\overline{\mathrm{x}}$ ± SD, *n* = 10(T,H), *n* = 5(S))

The commercial enzymes used in this study originated from both microbial, animal and plant sources. Protamex (Novozymes A/S) is a microbial protease complex produced by the bacteria *Bacillus subtilis*. The optimal conditions for maximal activity of Protamex has been reported as pH = 5.5–7.5 in the temperature range 35–60 °C, with inactivation at 85 °C [9, 23]. Corolase PP (AB Enzymes) is a protease complex of animal origin, produced from pig (*Sus scrofa domesticus*) pancreas glands. The protease complex consists of several digestive enzymes with both endopeptidase and exopeptidase activity. Corolase PP exhibit optimal activity at pH = 8–9 and a temperature of 45 °C [9, 24]. Papain and Bromelain (Enzybel International) are both protease complexes of plant origin, extracted from the fruit of papaya trees (*Carica papaya)* and the stem and fruit of pineapple plants (*Ananas comosus*) respectively. Both protease complexes have maximal activity in pH 5–9, with Papain being more active at higher temperatures (40–80 °C) than Bromelain (20–70 °C) [25, 26]. Adding commercial enzymes significantly influenced the dry matter yield in the hydrolysate. Addition of Protamex and Papain+Bromelain gave a significantly (Two-sample T(18) = 32.6–35.7, p<0.05) higher dry matter yield in the hydrolysate (HwHPr: 4.9 ± 0.4 g/ 100 g raw material, HwHP+B: 5.2 ± 0.5 g/ 100 g raw material) compared to autolysis (HwHA: 1.5 ± 0.2 g/ 100 g raw material). For enzymatic hydrolysis of raw material Hw/o, all added commercial enzymes significantly (Two-sample T(18) = 10.5–37.2, p<0.05) increased the dry matter yield in the hydrolysate (Hw/oHPr, Hw/oHPP, Hw/oHP + B: 6.2 ± 0.6, 4.5 ± 1.2, 5.5 ± 0.4 g/ 100 g raw material respectively) compared to autolysis (Hw/oHA: 1.5 ± 0.2 g/ 100 g raw material). This indicates that addition of commercial enzymes is effective for maximizing the dry matter yield in the hydrolysate, consequently increasing the content of solubilized protein in this fraction. No significant difference was found between the dry matter yield in hydrolysate from autolysis compared to the zero sample (HwH0: 1.3 ± 0.3 g/ 100 g raw material, Hw/oH0: 1.6 ± 0.1 g/ 100 g raw material). This indicates that the endogenous enzyme activity does not contribute to solubilizing proteins in the hydrolysis process. Viscera from chicken is assumed to contain large amounts of endogenous proteases, with a maximal activity at pH = 2.5 and a minimal activity at pH = 6.5–7.0 [27]. This will result in a low activity at the physiological pH conditions of the enzymatic hydrolysis (Hw: pH = 5.8, Hw/o: pH = 5.5), which can explain the low dry matter yield in the hydrolysate from autolysis. These results are consistent with findings of low endogenous activity in the raw material, and a corresponding low dry matter yield in the hydrolysate, for enzymatic hydrolysis of mechanically deboned chicken [28]. Endogenous enzyme activity was on the other hand found to be high during enzymatic hydrolysis of rest raw material from cod (*Gadus morhua*) [10, 29]. The observed lower activity in raw material from egg-laying hens can be advantageous for the hydrolysis process, which will be discussed further in chapter 3.2.2.

### Quality of obtained fractions

#### Oil fraction

##### Thermal treatment

Thermal treatment was conducted with the aim of separating lipid from the rest of the components of the raw material. Based on the dry matter yield (Table 2), 58.4% (HwT) and 50.1% (Hw/oT) of the total lipid content of the raw material were recovered in the oil fraction after processing.

Oil from thermal treatment of both raw materials was of high quality and had a low oxidation status based on FFA content (≤ 0,4%) and TOTOX (≤ 3,9) (Table 3). This indicates that oil separated from egg-laying hens is stable against oxidation during processing at high temperatures (100 °C). Thermally separated oil from raw material Hw had a significantly (Two-sample T(18) = 8.6, p<0.05) higher TOTOX (3.9 ± 1.2) compared to the raw material Hw/o (1.1 ± 0.7). This can be explained by different composition of raw materials, indicating that viscera in raw material Hw could be a source of prooxidants like hemoglobin and/or endogenous lipases.

**Table 3:** Oxidative status of oils after processing

Sample	FFA (%)	PV (meq/kg)	AV	TOTOX
Thermal treatment				
HwT	0.41 ± 0,03	1.36 ± 0,54	1.19 ± 0,89	3.92 ± 1.17
Hw/oT	0.30 ± 0,02	0,49 ± 0,42	0.11 ± 0,24	1.10 ± 0.65
Enzymatic hydrolysis				
Hw0	0.36 ± 0.03	0.31 ± 0.44	*	
HwA	0.41 ± 0.04	0.39 ± 0.27	0.20 ± 0.69	0.99 ± 0.79
HwHPr	0.40 ± 0.03	0.91 ± 0.56	*	
HwHPP	0.61 ± 0.04	0.33 ± 0.21	*	
HwHP+B	0.48 ± 0.06	0.45 ± 0.21	*	
Hw/o0	0.29 ± 0.00	0.39 ± 0.32	*	
Hw/oA	0.36 ± 0.03	0.20 ± 0.32	*	
Hw/oHPr	0.29 ± 0.04	0.96 ± 0.60	*	
Hw/oHPP	0.97 ± 0.09	0.74 ± 0.44	*	
Hw/oHP + B	0.40 ± 0.03	0.97 ± 1.20	*	
Silaging				
HwSA	0.40 ± 0.02	18.70 ± 0.91	27.88 ± 1.00	65.27 ± 1.64
HwSA.Su	0.51 ± 0.02	7.68 ± 0.14	13.45 ± 1.35	28.80 ± 1.37
HwSF	0.49 ± 0.02	7.29 ± 0.14	3.35 ± 0.20	17.93 ± 0.28
HwSF.Su	0.55 ± 0.05	1.97 ± 0.23	9.30 ± 0.10	13.24 ± 0.34
Hw/oSA	0.28 ± 0.04	25.76 ± 0.23	23.85 ± 0.24	75.38 ± 0.41
Hw/oSA.Su	0.37 ± 0.03	14.82 ± 0.60	42.15 ± 0.27	71.79 ± 0.88
Hw/oSF	0.37 ± 0.03	31.71 ± 0.53	15.84 ± 0.84	79.26 ± 1.12
Hw/oSF.Su	0.38 ± 0.02	11.72 ± 0.19	17.29 ± 1.06	40.74 ± 1.09

Results from analysis of the oil separated from hens with viscera, head and feathers (Hw) and hens without viscera, head and feathers (Hw/o) by thermal treatment (T), enzymatic hydrolysis (H) and silaging (S). Abbreviations are given for the individual treatments: without hydrolysis (0), autolysis (A), 0,1% Protamex (Pr), 0,1% Corolase PP (PP), 0,1% Papain + Bromelain (P + B), acetic acid (A), acetic acid + 0,1% sulfite (A.Su), formic acid (F) and formic acid + 0,1% sulfite (F.Su). FFA, PV, AV and TOTOX are given ($\overline{\mathrm{x}}$ ± SD, *n* = 8(T,H), *n* = 4(S)). * Invalid results

The fatty acid composition of the oil (Table 4) was found to depend on the composition of the raw material, but not on the processing method used for separation. The dominating fatty acids in oils from both raw materials were palmitic acid (C16:0), oleic acid (C18:1 ω9) and linoleic acid (C18:2 ω6). Both raw materials contained high amounts of MUFA (> 45%). MUFA is well known for health beneficial effects, including lowering LDL cholesterol, blood pressure, blood glucose and lipid levels, all risk factors for developing cardiovascular diseases [30]. MUFA has also been found to have a protective effect on pancreatic β-cells, responsible for normal insulin storage and release. The function of these cells is impaired in humans suffering from diabetes mellitus [31]. Oils separated from raw material Hw generally had a higher content of MUFA (47.22 ± 0.1%), and a lower content of PUFA (29.7 ± 0.01%) and ω3 fatty acids (1.4 ± 0.0%), compared to oils from raw material Hw/o (MUFA: 45.5 ± 0.0%, PUFA 31.6 ± 0.0%, ω3: 1.8 ± 0.0%).

**Table 4:** Fatty acid composition in oils after processing

Fatty acid	HwT	Hw/oT
C14:0	0.6 ± 0,0	0,68 ± 0,00
C14:1	0.1 ± 0,0	0,07 ± 0,00
C15:0	0.1 ± 0,0	0,18 ± 0,00
C16:0	18.5 ± 0.1	16.4 ± 0.0
C16:1 ω9	0.7 ± 0.0	0.6 ± 0.0
C16:1 ω7	2.8 ± 0.0	3.1 ± 0.0
C17:0	0.1 ± 0.0	0.2 ± 0.0
C17:1	0.1 ± 0.0	0.2 ± 0.0
C18:0	3.6 ± 0.1	5.2 ± 0.0
C18:1 ω9/ ω11	41.8 ± 0.1	39.4 ± 0.0
C18:1 ω7	1.3 ± 0.0	1.6 ± 0.0
C18:2 ω6	28.1 ± 0.0	29.5 ± 0.0
C18:3 ω6	0.1 ± 0.0	0.1 ± 0.0
C18:3 ω3	1.3 ± 0.0	1.6 ± 0.0
C18:4 ω3	0.1 ± 0.0	0.1 ± 0.0
C20:0	0.1 ± 0.0	0.1 ± 0.0
C20:1 ω9/ ω11	0.1 ± 0.0	0.1 ± 0.0
C20:1 ω7	0.3 ± 0.0	0.5 ± 0.0
C20:2 ω6	0.1 ± 0.0	0.1 ± 0.0
C20:3 ω6	0.0 ± 0.0	0.1 ± 0.0
C20:4 ω6	0.0 ± 0.0	0.0 ± 0.0
C20:3 ω3	0.0 ± 0.0	0.0 ± 0.0
C20:4 ω3	0.0 ± 0.0	0.0 ± 0.0
C20:5 ω3	0.0 ± 0.0	0.0 ± 0.0
C22:0	0.0 ± 0.0	0.0 ± 0.0
C22:1 ω11	0.0 ± 0.0	0.0 ± 0.0
C22:1 ω9	0.0 ± 0.0	0.0 ± 0.0
C22:5 ω3	0.0 ± 0.0	0.0 ± 0.0
C24:0	0.0 ± 0.0	0.0 ± 0.0
C22:6 ω3	0.0 ± 0.0	0.0 ± 0.0
C24:1 ω9	0.0 ± 0.0	0.0 ± 0.0
Sum fatty acid		
Saturated (SFA)	23.1 ± 0.2	22.9 ± 0.0
Monounsaturated (MUFA)	47.2 ± 0.1	45.5 ± 0.0
Polyunsaturated (PUFA)	29.7 ± 0.1	31.6 ± 0.0
Omega-3 (ω3)	1.4 ± 0.0	1.8 ± 0.0

Fatty acid composition in oils from thermal treatment of hens with viscera, head and feathers (HwT) and hens without viscera, head and feathers (Hw/oT). The fatty acids are named by the ω-system, were Cx:y ωz denotes a fatty acid with a chain length of x carbon atoms containing y numbers of double bonds. z denotes the position of the first double bond, counting from the methyl end of the carbon chain. Content of the individual fatty acids are given as % of total fatty acids ($\overline{\mathrm{x}}$ ± SD, *n* = 2)

Rest raw material from egg-laying hens had a low content of ω3 fatty acids compared to marine rest raw material. ω3 fatty acids are associated with several beneficial health effects [6, 7]. This limits the application of oil from egg-laying hens as dietary supplements. A higher degree of unsaturation will, on the other hand, increase the oxidation rate [32]. Oil from egg-laying hens might therefore be more resistant to oxidation and find application areas where marine oils are less suited.

##### Enzymatic hydrolysis

Enzymatic hydrolysis of the raw material gave three fractions, where separated oil and hen protein hydrolysate (HPH) are the products of interest. The dry matter yield in the oil fraction (Table 2) corresponds to 59–82% (HwH) and 51–69% (Hw/oH) lipid recovery after processing of the raw material. This means that processing by enzymatic hydrolysis resulted in an equal or better oil separation compared to processing by thermal treatment. Similar to thermal treatment, the oil from enzymatic hydrolysis was found to be of high quality (Table 3: FFA ≤ 1,0 ± 0.1, TOTOX ≤1.0 ± 0.8). For all samples except HwHA, analysis of AV gave invalid values. This might be explained by the analysis method itself, which has shown to be inaccurate for values ≤89 [33]. Combined with the low PV values obtained (Table 3), this indicates that the oil from enzymatic hydrolysis also have a low oxidative status.

##### Silaging

The different treatments and the composition of the raw material influences oil fraction yield after silaging. Silaging of raw material Hw gave a significantly (Two-sample T(8) = 7.5, p<0.05) higher dry matter yield (Table 2) in the oil fraction (14.3 ± 1.3–17.7 ± 0.7 g/ 100 g raw material), compared to raw material Hw/o (2.6 ± 1.7–8.0 ± 2.0 g/ 100 g raw material). 53.1–65.7% of the total lipid content of the raw material was recovered in the oil fraction after processing of Hw, formic acid giving a higher recovery compared to acetic acid. This indicates that endogenous enzymes are active under the silaging conditions, and important in separating the different components of the raw material. Raw material Hw is likely to have a higher content of endogenous enzymes, due to the viscera content, which can explain the higher oil yield. Maximizing oil separation is favorable when silaging, because oxidation of lipids is likely to occur and can reduce the quality of the protein rich fraction [13]. In consequence, silaging is probably better suited for processing of raw material including viscera, head and feathers (Hw).

**Fig. 2:**
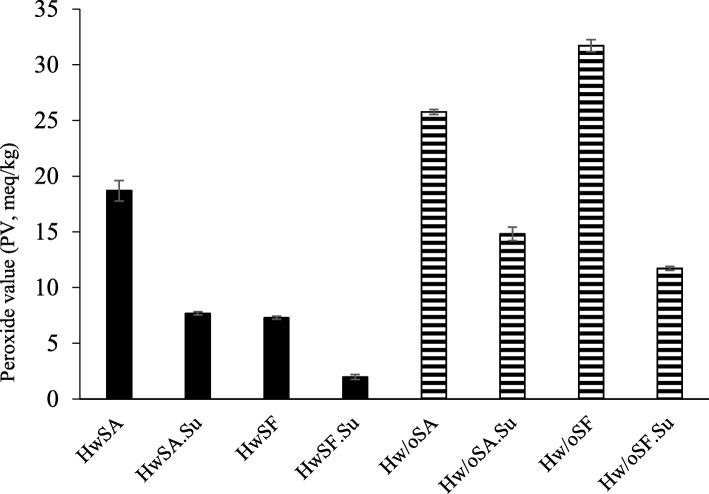
Oxidative status of oils. Peroxide value (meq/ kg oxygen) in oils separated from hens with viscera, heads and feathers (HwS, plain) and hens without viscera, head and feathers (Hw/oS, striped) by silaging ($\overline{\mathrm{x}}$ ± SD, *n* = 4). Abbreviations are given for the four individual treatments: acetic acid (A), acetic acid + 0,1% sulfite (A.Su), formic acid (F) and formic acid + 0,1% sulfite (F.Su)

The separated oil from all samples after silaging was found to be more oxidized compared to oil obtained after thermal treatment and hydrolysis, with TOTOX values ranging from 13.2–80.3 (Table 3). Silaging with formic acid gave significantly (Two-sample T(8) = 18.6, p<0.05) lower TOTOX values in the separated oil, compared to silaging with acetic acid, for raw material Hw. These results indicate that formic acid is more suited for silaging of egg-laying hens, compared to acetic acid. Sulfite was found to act as an antioxidant. The antioxidative effect becomes especially clear when looking at results from analysis of PV (Fig. 2). Adding sulfite before silaging significantly (Hw: Two-sample T(6) = 29.2–49.5, p<0.05, Hw/o: Two-sample T(6) = 42.0–87.2, p<0.05) reduced the PV of all samples. This indicates that addition of sodium bisulfite reduces the oxidation rate of the lipids in the raw material during silaging, and might therefore improve the quality of the silage. Silaging of Hw generally resulted in lower PV-values compared to Hw/o, again confirming the significance of endogenous enzymes to obtain optimal results in silaging.

The FFA content of the separated oil was found to be lower for raw material Hw/o compared to raw material Hw (Table 3) This indicates that endogenous enzymes contribute to hydrolyzing triacylglyserol in the silaging process, resulting in an increase in the FFA content. Nevertheless, the oils separated from the raw material by silaging was found to have low levels of FFA (≤ 0.6 ± 0.1) in general. Raw material Hw is thus probably best suited for processing by silaging, despite the increased FFA content, based on the better oil separation and the significantly lower TOTOX values as mentioned earlier.

#### Protein fraction

Hen protein hydrolysate (HPH) is the main product of enzymatic hydrolysis, and consist mainly of water-soluble proteins. Good quality hydrolysates are characterized by a high protein content and a low lipid content [10].

**Fig. 3:**
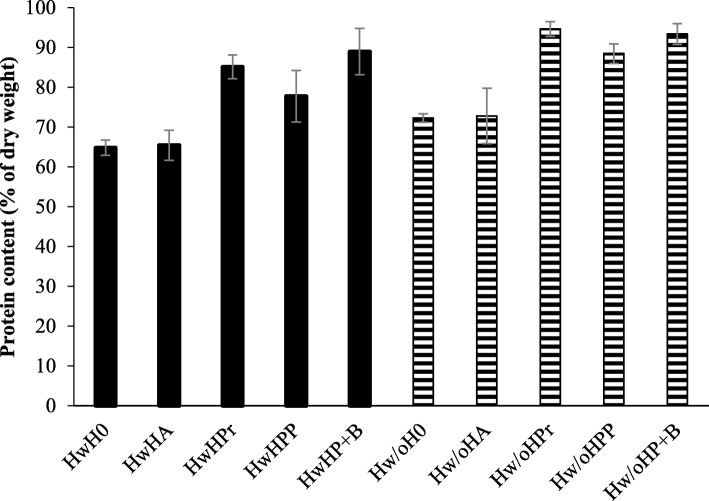
Protein content in hydrolysates. Protein content (% of dry weight) in hydrolysate from enzymatic hydrolysis of hens with viscera, head and feathers (HwH, plain) and hens without viscera, head and feathers (Hw/oH, striped) ($\overline{\mathrm{x}}$ ± SD, *n* = 3). Abbreviations are given for the five individual treatments used: without hydrolysis (0), autolysis (A), 0,1% Protamex (Pr), 0,1% Corolase PP (PP) and 0,1% Papain + Bromelain (P + B)

Addition of commercial enzymes increased the protein content of the HPH from 65.4 ± 3.7–72.8 ± 7.0% (Hw) to 85.1 ± 3.0–94.6 ± 1.8% (Hw/o) for enzymatic hydrolysis of both raw materials (Fig. 3). The increase was significant (Two-sample T(4) = 5.5–8.2, p<0.05) for hydrolysis with Protamex and Papain+Bromelain compared to autolysis. Protein recovery in HPH (Table 5) correspondingly increased from 5% to 11–25% for raw material Hw and from 6% to 22–32% for raw material Hw/o by addition of commercial enzymes.

**Table 5:** Protein recovery and degree of hydrolysis in hydrolysates after processing

Sample	Protein recovery (% of protein content of the raw material)	Degree of hydrolysis (DH, %)
Hw*H0*	4.5	42.2 ± 0.0
Hw*HA*	5.2	46.9 ± 0.0
Hw*HPr*	22.5	37.5 ± 0.5
Hw*HPP*	10.9	39.1 ± 0.4
Hw*HP + B*	25.0	32.7 ± 0.4
Hw/o*H0*	6.2	35.2 ± 2.3
Hw/o*HA*	5.9	30.9 ± 1.4
Hw/o*HPr*	31.7	24.3 ± 0.3
Hw/o*HPP*	21.8	26.4 ± 0.5
Hw/o*HP + B*	28.0	21.1 ± 0.5

Protein recovery (% of total protein content of the raw material) and degree of hydrolysis (%) in HPH from enzymatic hydrolysis of hens with viscera, head and feathers (HwH) and hens without viscera, head and feathers (Hw/oH) ($\overline{\mathrm{x}}$ ± SD, *n* = 3). Abbreviations are given for the five individual treatments used: without hydrolysis (0), autolysis (A), 0,1% Protamex (Pr), 0,1% Corolase PP (PP) and 0,1% Papain + Bromelain (P + B)

There was no significant increase in hydrolysis yield during autolysis. Both raw materials had a low dry matter yield (Table 2) in HPH from autolysis (1.5 ± 0.2%) compared to hydrolysis with commercial enzymes (4.5 ± 1.2–6.2 ± 0.6%). These results indicate that the proteolytic activity of endogenous enzymes is low under the conditions of the enzymatic hydrolysis. A low endogenous activity in the raw material can be beneficial for the hydrolysis process. To achieve a fully controlled hydrolysis, it might be necessary to terminate endogenous enzyme activity prior to addition of commercial enzymes [10]. This can be done by heat inactivation, where the raw material is subjected to high temperatures. On the other hand, high temperatures can negatively affect the protein yield in the hydrolysate due to denaturation and formation of protein-lipid complexes, which are less accessible for the proteases [10, 34, 35]. In raw material with low endogenous activity, initial heat inactivation can be excluded without endogenous enzymes affecting the control over the process. Unlike endogenous enzymes, commercial enzymes seem to be active under the conditions of the enzymatic hydrolysis based on the increase in protein content and protein recovery in HPH (Table 5). Commercial enzymes should therefore be added in enzymatic hydrolysis of both raw materials.

Addition of commercial enzymes significantly (Hw: Two-sample T(2) = 19.2, p<0.05, Hw/o: Two-sample T(2) = 8.2, p<0.05) decreased the lipid content in HPH, from 0.9 ± 0.0–1.9 ± 0.0% to 0.3 ± 0.0–1.1 ± 0.0%, for all samples except Hw*HPP*. This indicates that Corolase PP is not as effective for enzymatic hydrolysis of the raw material as are Protamex and Papain + Bromelain. The assumption was confirmed by the analysis of the protein content (Fig. 3), which was found to be lower in HPH from hydrolysis with Corolase PP compared to hydrolysis with the two other commercial enzymes. Freeze dried HPH from Hw*HPP* also had an apparent darker color, which can be explained by formation of dark pigments (melanoidins) through the Maillard reaction. Lipid oxidation products can act as precursors in the reaction, and an increased lipid content therefore makes HPH more susceptible to darkening. This is an undesired outcome, as it might influence the color of the food product if HPH were to be added as a functional ingredient [34, 36].

The molecular weight distribution of peptides in HPH from autolysis of both raw materials was found to be narrow, mainly consisting of peptides < 500 Da. This equals small oligopeptides and free amino acids, which are likely to originate from the raw material prior to processing. The raw material will at any time contain water-soluble proteins, and one explanation might therefore be that these were “washed out” during the pre-hydrolytic steps of the processing. The high share of peptides with a low molecular weight in HPH from autolysis was also reflected in the DH (Table 5: Hw*HA*: 46.9 ± 0.02%, Hw/o*HA*: 30.9 ± 1.4%), which was found to be significantly higher (Hw: Two-sample T(3) = 38.3, p<0.05, Hw/o: Two-sample T(3) = 5.3, p<0.05) in HPH from autolysis compared to HPH from hydrolysis with commercial enzymes (21.1 ± 0.5–39.1 ± 0.4%). No significant increase was observed between the weight distribution of peptides in HPH from the zero-samples and autolysis (Hw: Two-sample T(2) = 2.2, p<0.05, Hw/o: Two-sample T(2) = 1.8, p<0.05). This again strengthens the assumption that endogenous enzymes are not involved in solubilizing protein during the hydrolysis process. Based on the higher DH in HPH from autolysis (Hw*HA*: 46.9 ± 0.0) compared to the zero-sample (Hw*H0*: 42.2 ± 0.0) (Table 5), some protease activity might have occurred during hydrolysis of raw material Hw. This might indicate that the endogenous enzyme activity contributes to break down the water-soluble peptides that are already present prior to processing, and also confirms the higher endogenous enzyme activity of raw material Hw compared to raw material Hw/o.

**Fig. 4:**
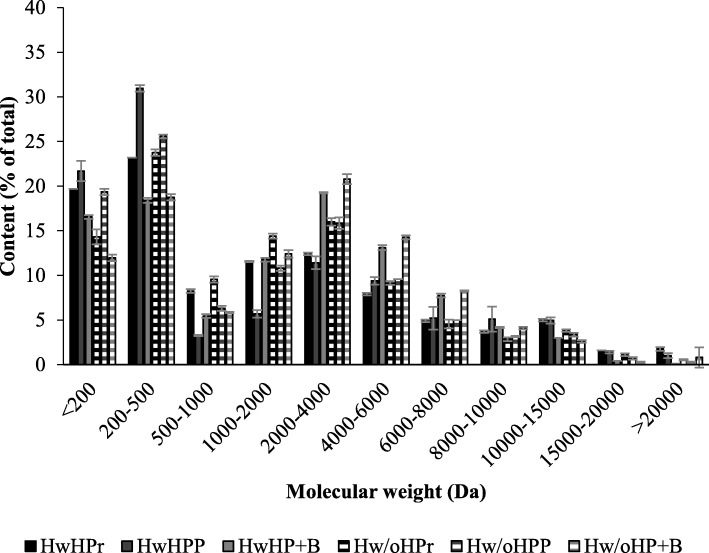
Molecular weight distribution of peptides in hydrolysates. Molecular weight distribution (Da) of peptides in freeze dried hydrolysate from enzymatic hydrolysis of hens with viscera, head and feathers (HwH, plain) and hens without viscera, head and feathers (Hw/oH, striped). Abbreviations are given for the individual treatments used: 0,1% Protamex (Pr), 0,1% Corolase PP (PP) and 0,1% Papain + Bromelain (P + B). Peptide content in the different weight intervals are given as % of total protein content ($\overline{\mathrm{x}}$ ± SD, *n* = 2)

Hydrolysis with added commercial enzymes gave HPH with peptides of a much broader weight distribution, and an increase in the content of peptides with higher molecular weight, as shown in Fig. 4.

Use of commercial enzymes significantly increased the protein content of the HPH (Fig. 3), as described earlier. This leads to an increased percentage of peptides with a higher molecular weight in HPH, and thereby a reduction of the percentage contribution of the smaller peptides. An increased percentage of high molecular weight peptides will in turn result in a lower DH in HPH from hydrolysis with commercial enzymes compared to autolysis (Table 5). These results contribute to strengthen the hypothesis that commercial enzymes are effective in solubilizing the protein content of raw material from egg-laying hens. The molecular weight distribution was also found to be dependent on type of commercial protease added. Papain + Bromelain gave a significantly (Hw: Two-sample T(2) = 15.6, p<0.05, Hw/o: Two-sample T(2) = 9.6, p<0.10) higher percentage of peptides in the weight interval 2000–4000 Da (19.3–20.8%) and a significantly (Hw: Two-sample T(2) = 24.0, p<0.05, Hw/o: Two-sample T(2) = 14.1, p<0.05) lower percentage of peptides in the weight interval 200–500 Da (18,4–18,8%) compared to Protamex and Corolase PP (2000–4000 Da: 11.4–16.0%, 200–500 Da: 23.2–40.0%). This indicates that treatment with Protamex and Corolase PP results in a higher degree of hydrolysis of peptides compared to Papain + Bromelain, confirmed by the results from analysis of DH (Table 5). HPH from hydrolysis with Papain + Bromelain is therefore likely to have better functional properties (like water holding capacity, emulsification, ability to form stable gels), while at the same time having an increased risk of bitterness [36, 37].

### Digestibility

Processing by silaging turns the raw material into a viscous sludge (silage) by the activity of endogenous digestive enzymes. Success of application in feed will significantly be defined by the digestibility of the raw material. The pepsin digestibility of defatted silage was found to be 63.2 ± 3.0–79.7 ± 1.7% (Table 6). For a protein source to be of good quality, the digestibility should be approximately that of milk protein (casein) or egg protein (albumin) [34]. Silaging increased the digestibility compared to that of the raw material prior to processing (57.3 ± 1.6–66.2 ± 2.0%). The increase in digestibility was found to be significant (HwSA.Su: Two-sample T(2) = 7.3, p<0.05, Hw/oSA: Two-sample T(2) = 7.1, p<0.05) for samples HwSA.Su and Hw/oSA, compared to raw material Hw and Hw/o respectively. This might indicate that acetic acid is the best treatment for silaging to obtain silage with the highest digestibility. Considering the small difference in digestibility between samples added formic acid compared to acetic acid for raw material Hw, formic acid is nevertheless likely to be the best treatment based on dry matter yield in the oil fraction and TOTOX values. The results from pepsin digestion is promising for the use of silage as a protein source in animal feed, even though it was found to be lower compared to digestibility of casein, which was found to be 83.9 ± 0.1 in this study.

**Table 6:** Protein digestibility of silage after processing

Sample	Pepsin digestibility (%)
Casein	83.9 ± 0.1
Raw material Hw	66.2 ± 2.0
Raw material Hw/o	57.3 ± 1.6
Hw*SA*	69.9 ± 1.6
Hw*SA.Su*	79.7 ± 1.7
Hw*SF*	72.6 ± 0.8
Hw*SF.Su*	70.2 ± 2.0
Hw/o*SA*	72.5 ± 2.6
Hw/o*SA.Su*	74.9 ± 4.6
Hw/o*SF*	64.0 ± 3.4
Hw/o*SF.Su*	63.2 ± 3.0

Pepsin digestibility (%) of silage from silaging of hens with viscera, heads and feathers (HwS, plain), hens without viscera, head and feathers (Hw/oS, striped), raw material Hw, raw material Hw/o and casein ($\overline{\mathrm{x}}$ ± SD, n = 2). Abbreviations are given for the four individual treatments: acetic acid (A), acetic acid + 0,1% sulfite (A.Su), formic acid (F) and formic acid + 0,1% sulfite (F.Su)

### Evaluation of technological processing method

This study identified three technological processing methods suitable for utilization of the lipid and protein content in discarded egg-laying hens: thermal treatment, enzymatic hydrolysis and silaging. Both thermal treatment and silaging are simple and low-cost processing methods that has a long tradition of use for processing of animal rest raw material [8, 12]. Microbial growth is restricted by heat and low pH respectively. The simplicity of the processing and equipment associated with these processing methods also makes it possible to handle large amounts of rest raw material, which is often a requirement for the technology to be applicable in an industrial context. Enzymatic hydrolysis is a more complex and expensive processing method, that has been suggested as a good option to produce products for human consumption [10]. In Norway, production of ingredients for human consumption falls under different regulatory frameworks than ingredients for animal feed. This puts stricter requirements on processing conditions, quality of the raw material and the processing products. The mild processing conditions of enzymatic hydrolysis are ideal for human consumption, but also puts less restrain on microbial growth and thus require both quality of raw material and hygiene to be optimal. The complexity of the processing equipment might also provide an obstacle to industrial use, in addition to the cost of enzymes. The results of this study has shown the potential of enzymatic hydrolysis to utilize protein and lipid from discarded egg-laying hens for human consumption. Thermal treatment was also found to produce oil that meet the quality requirements of food products. Silaging under the processing conditions used for this study does not produce products that can be used for human consumption, but as with the protein fraction from thermal treatment, can be applied for animal feed.

## Conclusion

This study has shown that thermal treatment, enzymatic hydrolysis and silaging can be used for processing of discarded egg-laying hens in order to utilize the protein and lipid content of the raw material. Thermal treatment at 100 °C can be used for production of good quality oil (≥ 58% yield) with low oxidative status but is not suited for solubilization of proteins. Enzymatic hydrolysis with Protamex, Papain, Bromelain and Corolase PP enables utilization of both the protein and lipid content of discarded egg-laying hens. Up to 32% of the protein can be solubilized in HPH, while up to 82% of lipids can be separated. The oil fraction of both thermal treatment and enzymatic hydrolysis was of good quality, with a low oxidative status, and can be used for human consumption. In addition, enzymatic hydrolysis yielded a protein fraction suitable for human consumption. However, compared to thermal treatment, this processing method is more comprehensive and expensive. Silaging with formic and acetic acid can be used to increase the digestibility of the protein fraction from 57 to 80%, which makes it more suitable for animal feed. The oil separated by silaging was of low quality, which limits the usage to technical purposes.

## Data Availability

The dataset used and/or analyzed during the current study are available from the corresponding author on reasonable request.

## Abbreviations

Hw: Hens with viscera, head and feathers
Hw/o: Hens without viscera, head and feathers
T: Thermal treatment
E: Enzymatic hydrolysis
A: Autolysis
Pr: Addition of 0.1% Protamex
PP: Addition of 0.1% Corolase PP
P + B: Addition of 0.1% Papain + Bromelain (1:1)
S: Silaging
A: Addition of acetic acid
F: Addition of formic acid
Su: Addition of sodium bisulfite
